# Current epidemiological HIV/AIDS situation in Republic of Moldova

**DOI:** 10.1186/1471-2334-14-S4-O1

**Published:** 2014-05-29

**Authors:** Lucia Pîrțînă, Angela Nagîț, Svetlana Popovici, Olga Staicova, Elena Golovco, Irina Cucerova, Iulian Oltu, Viorel Calistru

**Affiliations:** 1 Dermatovenerological and Communicable Diseases Hospital, Chişinău, Republic of Moldova

## 

In Republic of Moldova during 1987-2013, there were 8557 reported HIV+ cases, of which 2464 (28.8%) developed AIDS, 1752 (20.5%) died. Newly, 700-750 HIV positive cases are registered per year. The biggest number of HIV + is found at the age 25-39.

In relation to the way of transmission, the majority are infected by heterosexual contacts with tendency of increase, from 5.7% in 2000 to 91.4%. The next is among intravenous drug users (IDUs) with tendency of decrease from 83.7% in 2000 to 5% in 2013.

There are 5249 HIV+ persons in active evidence (77.13% of those who are alive), 3781 men and 3024 women.

Of these, 2493 persons, including 82 children are currently on antiretroviral treatment.

The incidence in 2013 was 17.99 per 100,000 population, in the eastern regions – 46.91.

The prevalence at January 1, 2014 constituted 173.43 per 100,000 population, in the eastern regions – 463.25. The large number of HIV newly infected cases in the east territories can be explained through limited access of the population to the prevention programmes, including population with high risk of infection.

Every year, there are registered about 80-90 cases of HIV infection among pregnant women. The rate of mother to child transmission in 2013 was 1.9%.

The Integrated Bio-Behavioral Study in key populations at higher risk was done in 2012-2013. From IDUs living in Chişinău, during the last year, 47.3% of the respondents took an HIV test and knew the result of the last test. HIV prevalence was 8.5% (16.4 in 2009).

From commercial sex workers living in Chişinău, during the last year 22.1% of respondents reported having taken an HIV test and knowing the result. HIV prevalence was 11.6% (6.1 in 2009).

From men who have sex with men, living in Chişinău, during the last year, 24.3% of the respondents took an HIV test and knew the result of the last test. HIV prevalence was 5.4% (1.7 in 2009).

The Republic of Moldova is classified as a concentrated/low prevalence country with a concentrated HIV epidemic in key populations such as injecting drug users and their regular sexual partners, commercial sex workers and men who have sex with men and there is an evidence of spread of the infection in the general population.

